# The dual role of neutrophils in sepsis-associated liver injury

**DOI:** 10.3389/fimmu.2025.1538282

**Published:** 2025-02-28

**Authors:** Lexin Fang, Yu Song, Jiangtao Chen, Yueping Ding

**Affiliations:** ^1^ Department of Intensive Care Unit, The Second Affiliated Hospital of Zhejiang Chinese Medical University, Xinhua Hospital of Zhejiang Province, Hangzhou, Zhejiang, China; ^2^ Department of Hepatology, The Second Affiliated Hospital of Zhejiang Chinese Medical University, Xinhua Hospital of Zhejiang Province, Hangzhou, Zhejiang, China

**Keywords:** immunosuppression, inflammation, liver injury, neutrophil, sepsis

## Abstract

Sepsis is often accompanied by liver injury and is associated with an increase in the number of circulating and hepatic neutrophils. In sepsis-associated liver injury, neutrophils exhibit phenotypic heterogeneity and perform both pro- and anti-inflammatory functions. Moreover, neutrophil dysfunction and neutrophil-associated immunosuppression are also involved in the pathogenesis of sepsis. Given the complex functionality of this cell type, the aim of this review was to describe the possible mechanistic role of neutrophils in sepsis-associated liver injury, with a brief introduction to neutrophil recruitment and subsequent discussion of the potential contributions of neutrophils to different subtypes of sepsis-associated liver injury.

## 1 Introduction

Sepsis is the main cause of death in intensive care units (ICU). It is accompanied by multi-organ dysfunction, with sepsis-associated liver injury occurring in 34–46% of patients with sepsis (1). Sepsis-associated liver injury (SALI) can generally be classified as either hypoxic hepatitis, cholestatic, hepatocellular, or severe cholangitis (2, 3), although the precise pathogenesis remains to be elucidated. Neutrophils are first-responder cells recruited to protect host organisms from infection or sterile tissue injury, and their accumulation has been observed in the livers of model animals with sepsis (4). Neutrophils clear pathogens through a variety of processes, including phagocytosis, degranulation, reactive oxygen species (ROS) production, and neutrophil extracellular traps (NETs), which consist of nuclear DNA, histones, and proteases. However, there is evidence that neutrophils are an independent predictor of SALI (5), and excessive accumulation or dysfunction of neutrophils may induce SALI (4). Therefore, the role of neutrophils is a double-edged sword. This mini-review aimed to discuss the role of circulating neutrophils as a component of innate immunity in sepsis, with a focus on possible mechanisms through which neutrophils induce liver injury in patients with the disease.

## 2 Recruitment of neutrophils in sepsis

### 2.1 Sepsis-induced release of large numbers of neutrophils into the circulation

In infectious states, cytokines such as granulocyte colony-stimulating factor (G-CSF) and granulocyte-macrophage colony-stimulating factor are released and subsequently activate multiple transcriptional mechanisms that promote granulopoiesis. Ioannou et al. discovered that G-CSF exposure shortens the lifespan of mature neutrophils, causing a disproportionate shift in neutrophil populations toward an immature phenotype in septic patient plasma; these changes primarily occur in the late granulocyte or maturation phase, with potential enhancement by extracellular histones (6). The same group also reported high G-CSF levels were associated with a poor prognosis, whereas the onset of sepsis was delayed when mice were pre-treated with G-CSF 24 hours before infection. In addition, G-CSF promotes neutrophil egress from bone marrow, primarily by promoting the activation of the C-X-C motif chemokine receptor (CXCR) 4/CXCR2 axis, skewing the balance towards CXCR2 (7, 8). Given the increased proportion of immature neutrophils in the general circulation, their cell counts could be useful biomarkers of sepsis, helping rule in and rule out the possibility of the disease with a certain specificity (9). One study demonstrated a differential enrichment of neutrophil subsets in patients with sepsis (10). Using flow cytometry, they roughly grouped neutrophil subsets into mature, immature, and others based on the expression of cluster of differentiation 10 (CD10). They further identified that CD10^−^CD177^+^ immature neutrophil subset showed reduced of oxidative burst capabilities as well as phagocytosis, and CD10^+^ mature subset with high level of programmed death ligand 1 (PD-L1) exhibited inhibition of T-cell proliferation. Meghraoui et al. reported that CD10^-^ CD64^+^CD16^low/-^ CD123^+^ neutrophils and CD10^-^CD64^+^PD-L1^+^neutrophils could facilitate the early diagnosis of sepsis (11). Moreover, Chen et al. discovered that Ly6G^+^Lta4h^+^Sort1^+^ neutrophil (Neu-3) levels are closely correlated with the occurrence of SALI in a mouse model (12). In recent years, research methods such as single-cell RNA sequencing (scRNA-seq) transcriptomics and proteomics have been used to analyze neutrophil heterogeneity, enriching our understanding of these cells and facilitating the exploration of their unique characteristics in sepsis.

### 2.2 Neutrophil migration in circulation

The ability of neutrophils to combat infection is dependent on their ability to first undergo migration to the infectious site. The phases of migration include release from the bone marrow, migration and rolling, adherence, and transmigration (13). Neutrophil migration is influenced by the concentration gradient of chemoattractant signals, and the cells respond to these signals hierarchically, mainly through the activation of G-protein-coupled receptors (GPCRs) (14). However, neutrophil chemotaxis becomes impaired and migration may even be reversed in sepsis (15). Ciupe et al. utilized mathematical models to demonstrate that the tightly regulated migratory behavior of neutrophils toward an infectious site can be altered with different concentrations of lipopolysaccharide (LPS) (16). More specifically, neutrophils treated with ultra-low doses of LPS or those exposed to LPS for extended periods might lose their ability to move up the chemotactic gradient, whereas high-dose LPS treatment enhanced their directional migration. Bao et al. reported that priming of neutrophils with LPS somewhat prevented the onset and progression of LPS-induced sepsis in a murine model (17). Impaired neutrophil chemotaxis is also associated with the internalization or desensitization of GPCRs (18). In a clinical study investigating neutrophil surface receptors, Seree-Aphinan et al. found that only the levels of CXCR2 correlated with sepsis, that a decrease in CXCR2 expression occurred in parallel with the peak of infectious activity and that this change could be used to differentiate sepsis from systemic inflammatory responses (19). In addition to neutrophil surface receptors, a large proportion of immotile neutrophils and high neutrophil mobility could each serve as an independent predictor of sepsis in patients with cirrhosis (20).

### 2.3 Neutrophil recruitment to the liver

The mechanisms underlying neutrophil recruitment to liver sinusoids differ from the classical recruitment cascade and appear independent of processes involving integrins and rolling, which have been extensively reviewed elsewhere (21). Neutrophils with high CXCR2 expression levels are recruited to the liver and guided along the concentration gradient of chemoattractant signals later in sepsis. In cases of endothelial barrier damage, immature neutrophils with low CXCR2 expression move to the liver for disposal through diapedesis (22). However, in comparison to septic mice who are fed high-fat and normal diets, neutrophil accumulation in the liver was shown to be unaffected by CXCR2, and obese mice exhibited a higher survival rate (23). Infections caused by different pathogens may induce differential neutrophil recruitment to the liver (24). For example, formyl peptide receptor signaling may act as an initial chemotactic signal during *Listeria monocytogenes* infection (25). In a septic model involving *Staphylococcus aureus* infection, heparan sulfate (HS) binding proteins were shown to be significantly enriched on the surface of the hepatic vasculature and were involved in modulating neutrophil recruitment (26). Reducing endothelial HS sulfation can selectively attenuate hepatic neutrophil infiltration and tissue damage. Pioneering neutrophils in the liver lead to swarming behavior via self-amplifying chemotactic signals, resulting in tissue damage (27, 28), with leukotriene B4 (LTB4) playing an important role in this process. Yu et al. demonstrated, in a cecal ligation and puncture (CLP)-induced murine model, that SALI and neutrophil infiltration could be ameliorated by inhibiting 5-lipoxygenase/LTB4 through caffeic acid administration (29). Neutrophil transmigration to the liver parenchyma occurs more rapidly compared to the migration rates toward other organs due to discontinuities in the hepatic sinusoidal capillaries (30), and neutrophil recruitment to the liver sinusoids is dependent on organ-specific adhesion mechanisms. Neutrophils adhere to the sinusoidal endothelium in the liver predominantly through interactions between a cluster of differentiation 44 (CD44) and hyaluronan (HA) as well as between dipeptidyl peptidase 1 (DPEP-1) and its ligands (31). Notably, the CD44–HA interaction is weak (32), although it is enhanced by the modification of heavy chains during sepsis, with the levels of heavy chain Itih3 in hepatic tissue samples increasing significantly (33). Furthermore, toll-like receptor 4 (TLR4) signaling affects serum-derived HA-associated protein, promoting the direct adherence of neutrophils to the sinusoidal endothelium of the liver partially through HA–CD44 interactions (34). McDonald et al. discovered that blocking HA–CD44 interactions using an anti-CD44 antibody significantly ameliorated LPS-induced hepatic injury (32). In addition, neutrophils promote their own extravasation through the endothelium into inflamed tissue by expressing macrophage-1 antigen (MAC-1) and very late antigen 4, which binds to intercellular adhesion molecule 1 (ICAM-1) and vascular cell adhesion molecule 1, respectively, on hepatic sinusoidal endothelial cells (21). It has been shown that LPS induces neutrophil priming, as reflected by increased ICAM-1 expression; this change is associated with enhanced neutrophil infiltration into the liver and tissue injury (35). Xiao et al. found that reducing hepatic neutrophil transmigration and hepatocyte injury through the administration of neutrophil membrane-mimicking nanodecoys in an endotoxemia mouse model may be linked to decreased endothelial ICAM-1 expression (36).

## 3 Effects of neutrophils on the liver during sepsis

### 3.1 Protective functions of neutrophils

Neutrophils act as a first line of defense against infections; once they enter the liver, they initiate various antimicrobial activities by secreting protein hydrolases and ROS. In the liver, neutrophils have a weak ability to entrap circulating bacteria; however, this ability is enhanced by the release of NETs (37). GPCRs modulate ROS levels to mediate the release of NETs in the early stages of disease (38). Neutrophils might tune their response according to a microbe’s size, as large pathogens promote the upregulated expression of interleukin (IL)-1β and neutrophil recruitment by triggering extracellular ROS release, while also inducing neutrophils to selectively release NETs (39, 40). Shao et al. reported that ‘targeted nuclear degranulation’ that a portion of the CD44 expressed on the surface of neutrophils moved to the nucleus after neutrophil activation was delayed to limit the rapid formation of NETs in response to strong stimulation in mouse models (41). Moreover, Oliveira-Costa et al. proposed the “innate triad” model, which involves interactions between neutrophils, platelets, and macrophages to enhance pathogen clearance (42).

Neutrophils are also associated with the resolution of liver inflammation and tissue repair. The restorative effects of neutrophils have been investigated in a murine model of toxic liver injury (43). Neutrophils may help induce a phenotypic switch in macrophages toward a pro-restorative profile through microRNA-223 or ROS, thereby resolving inflammation (44, 45). In addition, microRNA-223 is known to regulate neutrophil elastase (NE) enrichment to protect the liver from LPS-induced injury (46).

### 3.2 Neutrophil-driven hyperinflammation in liver injury

Overexuberant recruitment or the uncontrolled activation of neutrophils may lead to an overwhelming pro-inflammatory condition that is associated with multi-organ injury. An abundant neutrophil population has been observed in mouse livers during sepsis (30); however, it remains uncertain whether their lifespan in the liver can be extended or constantly replenished *de novo* from bone marrow. In recent years, granulocyte–monocyte progenitors have been identified to undergo release into the circulatory system during sepsis, and cell division has been reported in neutrophils (47). Therefore, further studies are required to investigate whether numerous neutrophils in the liver partially originate from peripheral neutrophil proliferation before altering their function. Several studies have utilized scRNA-seq to analyze the phenotype and function of hepatic neutrophils in SALI (12, 48). Chen et al. identified three distinct hepatic neutrophil subsets in septic mouse models (12) and demonstrated that the proportion of pro-inflammatory subsets continued to increase in a time-dependent manner. TLR plays an important role in the activation of neutrophils, driving a shift toward pro-inflammatory responses. One study also confirmed that microRNA-let-7b regulates neutrophil function by inhibiting the TLR4/nuclear factor kappa-B (NF-κB) pathway while also attenuating hepatic inflammation in septic mice, with the data showing decreased gene expression of tumor necrosis factor (TNF)-α and IL-8 in neutrophils (49). He et al. demonstrated that neutrophils identify pathogen-associated molecular patterns (PAMPs) via TLR2, which actives NF-κB signaling and the subsequent release of pro-inflammatory factors (48), and that artesunate treatment could reverse the increase in the proportion of pro-inflammatory subsets by inhibiting TLR2 expression while alleviating sepsis-induced liver injury in mouse models. Moreover, these proinflammatory neutrophils may be associated with dysregulated cytoplasmic Ca^2+^ concentrations and enhanced membrane depolarization and glycolytic metabolism (50, 51).

There is some evidence that the oxidative burst capacity of neutrophils is altered in SALI. Sustained neutrophil activation induces oxidative bursts by activating the GPCR/phospholipase C (PLC)/Ca^2+^ signaling pathway (14). Wang et al. reported that increased oxidative stress might result from activation of CXCR2 and its downstream target protein kinase C in neutrophils, with the process being regulated by CXCL2 on macrophage extracellular vesicles isolated from mouse (52). ROS activate the formation of NOD-like receptor 3 (NLRP3) via thioredoxin-interacting protein activation, subsequently resulting in IL-1β release into the extracellular space, causing an excessive inflammatory response (53). Moreover, NLRP3 triggers caspase-1-mediated endothelial pyroptosis, increasing hepatic vascular permeability and the likelihood of mortality (54). NETs exert a protective function by limiting bacterial spread; however, NET production or imbalances in clearance can also induce thrombosis, disseminated intravascular coagulation, and tissue damage (55). NET expression in neutrophils is upregulated in a time-dependent manner in septic liver tissues, far surpassing the levels in other areas of the microcirculation (56, 57). Hsieh et al. reported that histone H4 might trigger a sustained elevation of intracellular Ca^2+^ levels in human neutrophils, thereby inducing hydrogen peroxide production and degranulation through the G protein/phosphoinositide 3-kinase (PI3K) pathway to promote inflammatory responses (58). Levels of calgranulins S100A8/S100A9 become elevated in the blood of patients with sepsis and may be produced from neutrophil movement (59). Hepatic neutrophils with a hyperactivated phenotype exhibit high expression levels of S100A8/S100A9 in septic models (12), which may damage the liver by binding to receptors of advanced glycation endproducts (RAGE) and subsequently activating NF-κB signaling to promote TNF-α expression, providing evidence of its role as a damage-associated molecular pattern (DAMP) (4). Zhang et al. reported that S100A9 can disrupt mitochondrial respiratory chain functionality in the hepatic tissues of septic mice (60). S100A9 knockout ameliorated liver injury in these animals by inhibiting protein kinase B (Akt) and 5′adenosine monophosphate-activated protein kinase-activated mitochondrial metabolism.

### 3.3 Neutrophil-associated immunosuppression in liver injury

Although hyperactivated neutrophil responses lead to SALI, these cells exhibit impaired antimicrobial functions, and neutrophil-associated immunosuppression is reduced, implying an increased susceptibility to secondary infections. In a study involving septic mice in which neutrophilic gasdermin D (GSDMD) was specifically knocked out, no significant change in the degree of neutrophilic NET formation was observed, and this deletion led to more severe liver damage (61). This suggests that neutrophil-specific GSDMD may regulate the bactericidal activity of neutrophils and that impaired regulation is unrelated to NETs. Taylor et al. demonstrated that neutrophils exhibit substantially diminished functional capacity, including impairment of oxidative burst capabilities and phagocytosis in patients with SALI (62). The energetic state of neutrophils governs their function. In comparison to patients with sepsis and healthy volunteers, the lactic acid levels were lower in the septic patients, as evidenced by a study in which sustained LPS stimulation inhibited neutrophil phagocytosis through the decreased production of lactates and the inhibition of glycolysis via the PI3K/Akt/hypoxia-inducible factor (HIF)-1α pathway (63). A study identified an important role of monocyte chemotactic protein-induced protein-1 (MCPIP-1) in reducing the oxidative burst of neutrophils through degradation of cold-inducible RNA in mice with infectious hepatic disease in which neutrophilic MCPIP-1 expression becomes elevated (64).

Impaired antibacterial activity in neutrophils can also be attributed to their hyporesponsiveness to pathogens. Neutrophilic susceptibility to pathogens is also reduced by high levels of pro-inflammatory cytokines, soluble receptors, and endotoxins (65). The sustained inflammatory stimulation may be associated with tolerance development that affects TLR signaling or the upregulation of inhibitors of TLR signaling (66). Conversely, however, DAMP expression levels remain persistently elevated in LPS-challenged model piglets, which share similar pattern recognition receptors (PRRs) on neutrophils as well as microbial-associated molecular patterns, leading to impaired pathogen recognition (67, 68). Recent studies have identified a subset of low-density neutrophils (LDNs), including immature neutrophils and myeloid-derived suppressor cells with immunosuppressive characteristics, and there is evidence that some LDNs are degranulated from high-density neutrophils (HDNs) (69, 70). While these LDNs exhibit limited phagocytic abilities in a CLP mouse model, they are more actively engaged in the formation of NETs, which promote naïve CD4^+^ T cell differentiation into regulatory T cells (Tregs) while enhancing their immunosuppressive function (71). Neutrophil-derived immunosuppressive cells have been reported to undergo expansion in the liver of septic mice and are associated with T-cell dysfunction (72). Human neutrophils produce MAC-1 and PD-L1, which exert immunosuppressive effects that include T-lymphocyte apoptosis and the inhibition of T-cell activation and proliferation (73, 74). In addition, neutrophils in patients with sepsis produce large amounts of immunosuppressive cytokines, such as IL-10 (10).

### 3.4 The role of neutrophils in the gut–liver axis

Intestinal dysbiosis and disruption of the intestinal barrier induce intestinal bacteria and their metabolites translocation that causes the inflammatory response. Intestinal dysbiosis was associated with severe liver injury in septic models (75). Generally, intestinal pathogens and their products can go through the circulation, portal, and biliary to the liver and are processed by the liver (76). A review analyzing changes in neutrophil intracellular bacterial communities at different stages of sepsis found that the alterations in neutrophil-specific microbiomes were similar to intestinal microbiome composition (77). Intestinal epithelial cells and hepatocytes can produce LPS-binding protein, which enhances LPS transfer, binds to the membrane CD14 on neutrophils, and consequently promotes the inflammatory response (76). Liu et al. discovered that gut-derived bacteria and LPS promote the formation of NETs in the liver via TLR4 in a mice model (78). Recent studies have discovered that the intestinal microbiome can also regulate intrahepatic neutrophil infiltration Using *in vivo* imaging to track neutrophil movement in mice with Staphylococcus aureus infection, D-lactate-producing gut microbiota prime hepatic endothelial cells to upregulate DPEP-1 expression (79). Collectively, these studies suggest that targeted restoration of axis equilibrium to combat gut dysbiosis in SALI may prevent excessive neutrophil recruitment.

## 4 The role of neutrophils in different types of SALI

### 4.1 Neutrophils in sepsis-associated cholestatic liver injury

Sepsis-associated cholestasis is a clinical phenotype of SALI. A study that evaluated liver samples from patients with sepsis revealed that ductular cholestasis holds diagnostic value for identifying the disease, with a sensitivity of 68% and a specificity of 45% (3). Furthermore, hepatic neutrophilic infiltration has been observed, although there was no significant difference in the rates of portal and lobular neutrophilic inflammation in patients with and without sepsis (3). Accumulation of neutrophils around the bile ducts has been observed in an acute biliary epithelial cell mouse model (80). However, impaired LPS excretion by hepatocytes has been observed in bile salt efflux pump knockout mice; in these animals, LPS was not excreted through bile acids and was a direct cause of further infiltration of neutrophils and inflammatory mediators (81). Sepsis-associated cholestasis may be associated with impaired bile excretion rather than an increase in bile synthesis (82). Wu et al. reported a reduction in the direct IL-1β/IL-18-mediated neutrophilic damage to transporter proteins, which resulted in the restoration of tubular transporter protein and sepsis-induced hyper-bileacidaemia in NLPR3 knockout mice (83). In addition, septic bile duct-associated neutrophil subsets exist in an exhausted state, and the biological function is characterized by reduced neutrophil migration and phagocytosis, making it difficult to control infections and exacerbating septic shock development (84). Finally, a recent study demonstrated that tuft cells are negatively associated with biliary inflammation and microbiome-dependent neutrophilic infiltration, and biliary inflammation increased in tuft cell-deficient mice of cholestasis (85).

### 4.2 Neutrophils in sepsis-associated hypoxic liver injury

In sepsis, the liver increases its capacity for oxygen extraction; however, decreased hepatic perfusion and impaired oxygen utilization can still result in hepatocyte death (86). In addition, increased oxidative stress in septic livers is somewhat indicative of oxygen depletion, which ultimately leads to hypoxic liver injury. Neutrophils can work actively under hypoxic conditions and can sense oxygen tension; a series of responses triggered by stimulation is succeeded by increased degranulation, reduced ROS production, and prolonged survival (87, 88). However, continuous LPS stimulation has been shown to reduce glycolysis in neutrophils through the HIF-α pathway, thereby affecting their phagocytic and migratory functions (63). Recruited neutrophils accumulate in the sinusoids, blocking the lumen and exacerbating ischemia and hypoxia (89). Boufenzer reported that hypoxic conditions activate triggering receptors expressed on myeloid cell-1 (TREM-1), which synergizes with TLR4 to induce intracellular calcium currents and ROS production, thereby enhancing NET release by human and murine neutrophils (90). NETs impair hepatic microcirculation and further exacerbate hypoxia (91); in turn, ischemia and hypoxia lead to neutrophil recruitment to the liver in large numbers. The detrimental cycle of increased neutrophil accumulation and hypoxia can continue and accelerate liver injury progression.

## 5 Discussion

Neutrophils can be regarded as either a blessing or a curse, depending on their differential functionality in SALI. Proteases, ROS, and NETs released from neutrophils are involved in pathogens clearance and liver injury resolution, but they also act as pro-inflammatory mediators leading to liver injury. This may be influenced by time, concentration, and environment. This dual role of neutrophils deepens the difficulty of exploring their therapeutic role in SALI. By studying neutrophil heterogeneity, we have gained insight into the functions of different neutrophil subsets (**Table 1**), and helped develop targeted therapies against neutrophils and their associated components. Animal studies found that pro-inflammatory neutrophils are progressively increased during the progression of SALI, but the mechanism remains unclear. Reversing the proportion of pro-inflammatory neutrophils may be one of the future research directions.

**Table 1:** The characteristics of neutrophil subsets in sepsis.

Species	Subset	Phenotype	Function/clinical relevance	Reference
Human	Mature neutrophil	CD10^+^PD-L1^+^	facilitate diagnosis of sepsis	(10)
Human	Immature neutrophil	CD10^−^CD177^+^	lower level of ROS and phagocytic capacity	(10)
		CD10^-^ CD64^+^CD16^low/-^ CD123^+^	facilitate the early diagnosis of sepsis	(11)
Human/Mouse	Inhibitory neutrophil	PD-L1^+^	inhibition of T-cell activation and proliferation	(10, 11, 74)
Mouse	Low-Density Neutrophil		limited phagocytic abilities; promote Tregs and enhance their immunosuppressive function	(71)
Mouse	Neu-3	Ly6G^+^Lta4h^+^Sort1^+^	closely correlated with the occurrence of SALI	(12)

Neutrophils are first-responder cells recruited to infection. In recent years, progress has been made in the use of neutrophils as carriers in the development of drugs to treat SALI; however, because of the difficulty of obtaining liver specimens from septic patients, this research is still in the laboratory stage. Developing animal models that more simulate patients with SALI could help study potential treatments of SALI.
